# The Hyperactivity–Impulsivity–Irritiability–Disinhibition–Aggression–Agitation Domain in Alzheimer’s Disease: Current Management and Future Directions

**DOI:** 10.3389/fphar.2019.01109

**Published:** 2019-09-27

**Authors:** Rachel M. Keszycki, Daniel W. Fisher, Hongxin Dong

**Affiliations:** ^1^Department of Psychiatry and Behavioral Sciences, Northwestern University Feinberg School of Medicine, Chicago, IL, United States; ^2^Department of Psychiatry and Behavioral Sciences, University of Washington Medical Center, Seattle, WA, United States

**Keywords:** behavioral and psychological symptoms, dementia, Alzheimer’s disease, non-pharmacological treatment, pharmacological intervention

## Abstract

Behavioral and psychological symptoms of dementia (BPSD) afflict the vast majority of patients with dementia, especially those with Alzheimer’s disease (AD). In clinical settings, patients with BPSD most often do not present with just one symptom. Rather, clusters of symptoms commonly co-occur and can, thus, be grouped into behavioral domains that may ultimately be the result of disruptions in overarching neural circuits. One major BPSD domain routinely identified across patients with AD is the hyperactivity–impulsivity–irritiability–disinhibition–aggression–agitation (HIDA) domain. The HIDA domain represents one of the most difficult sets of symptoms to manage in AD and accounts for much of the burden for caregivers and hospital staff. Although many studies recommend non-pharmacological treatments for HIDA domain symptoms as first-line, they demonstrate little consensus as to what these treatments should be and are often difficult to implement clinically. Certain symptoms within the HIDA domain also do not respond adequately to these treatments, putting patients at risk and necessitating adjunct pharmacological intervention. In this review, we summarize the current literature regarding non-pharmacological and pharmacological interventions for the HIDA domain and provide suggestions for improving treatment. As epigenetic changes due to both aging and AD cause dysfunction in drug-targeted receptors, we propose that HIDA domain treatments could be enhanced by adjunct strategies that modify these epigenetic alterations and, thus, increase efficacy and reduce side effects. To improve the implementation of non-pharmacological approaches in clinical settings, we suggest that issues regarding inadequate resources and guidance for implementation should be addressed. Finally, we propose that increased monitoring of symptom and treatment progression *via* novel sensor technology and the “DICE” (describe, investigate, create, and evaluate) approach may enhance both pharmacological and non-pharmacological interventions for the HIDA domain.

## Introduction

Though dementia encompasses an array of neurodegenerative conditions and is characterized by a progressive decline in cognitive functions and ability to execute activities of daily living (Prince et al., 2013), severe behavioral and psychological symptoms of dementia (BPSD) are nearly universal in these patients. For instance, in Alzheimer’s disease (AD), which represents 60–80% of all dementias (Alzheimer’s Association, 2016), over 90% of patients display BPSD, including depression, anxiety, apathy, agitation and aggression, disinhibition, delusions, hallucinations, irritability and emotional lability, euphoria, and aberrant motor, sleep, and eating behaviors (Kales et al., 2019). These symptoms may present before clinically significant memory decline and are correlated with a greater likelihood of conversion to AD from mild cognitive impairment (MCI) (Serra et al., 2010). Overall, BPSD are associated with decreased quality of life, increased cognitive and functional decline, greater likelihood of institutionalization, and heightened risk of mortality (Gilley et al., 2004; Scarmeas et al., 2005; Wilson et al., 2006; Gaugler et al., 2009; Rowe et al., 2009). Moreover, these symptoms are correlated with greater direct and indirect costs as well as higher caregiver burden (Clyburn et al., 2000; Allegri et al., 2006; Herrmann et al., 2006).

Given the high prevalence of BPSD in neurodegenerative patients, clinical instruments to aid in BPSD assessment are essential. To date, there are more than 80 instruments for measuring BPSD (van der Linde et al., 2014b). Patients in the earlier stages of dementia may answer some of these measures *via* self-report, but as cognitive decline progresses, it is more common for clinicians, caregivers, or informants to complete these instruments based on their observations of patients’ BPSD (Conn and Thorpe, 2007). Of the measures available, the Neuropsychiatric Inventory (NPI) is most commonly used in both research and clinical settings (Conn and Thorpe, 2007; van der Linde et al., 2014b). The NPI provides information about the frequency and severity of patients’ overall BPSD and subdomains of symptoms (e.g., agitation and aggression) as well as an indication of caregiver distress. While the NPI was designed to assess BPSD across various forms of dementia, the Behavioral Pathology in AD Rating Scale (BEHAVE-AD) was specifically developed to measure global BPSD in patients with AD (Jeon et al., 2011; van der Linde et al., 2014b). The BEHAVE-AD is the second most cited assessment of global BPSD in the literature, and it provides information about the severity of a patient’s global and specific BPSD symptoms. Although measures of overall BPSD can provide insight into the presence and extent of specific symptoms, there are also numerous instruments designed to examine these in depth. Examples of symptom-specific measures include the Overt Aggression Scale (OAS) for irritability and aggression and the Cohen-Mansfield Agitation Inventory (CMAI) for agitation. Although these instruments were not originally created to assess these symptoms in patients with dementia, they are currently the most widely used instruments in the literature for measuring irritability, aggression, and agitation in the context of BPSD.

Clinical presentations of BPSD across patients vary widely due to baseline individual differences, dementia type, and severity of cognitive decline (Jeste et al., 2006; Petrovic et al., 2007; Azermai, 2015). Overall, however, BPSD tend to cluster into “domains,” as certain symptoms show high frequencies of co-occurrence (Jeste et al., 2006; Azermai, 2015). Indeed, numerous researchers and clinicians conceptualize symptom domains for BPSD (Jeste et al., 2006; Azermai, 2015) and have suggested that there is a high likelihood of common underlying molecular and cellular pathologies for symptoms in each domain (Aalten et al., 2003; Jeste et al., 2006; Aalten et al., 2007; Aalten et al., 2008). One systematic analysis of 62 studies utilizing unbiased clustering approaches, such as principal component, factor, latent class, or cluster analysis, on behavioral data from participants with dementia routinely identified specific BPSD domains across studies, including affective domain, apathy domain, psychosis domain, euphoria domain, and hyperactivity–impulsivity–irritiability–disinhibition–aggression–agitation (HIDA) domain (van der Linde et al., 2014a).

The HIDA domain, in particular, represents one of the most difficult sets of symptoms to manage in AD, accounts for much of the burden for caregivers and hospital staff, and represents an area of special concern regarding safety (Fuh et al., 2001; Rymer et al., 2002; Nguyen et al., 2008; Fauth and Gibbons, 2014). Aggression is correlated with an increased risk of self-injurious behavior (Gilley et al., 2004; de Jonghe-Rouleau et al., 2005). Impulsivity and impaired executive functioning are related to increased wandering and disorientation, which can in turn put patients at an increased risk for falls and mortality (Chiu et al., 2004). As a result of these risks and their impact on caregivers, patients with severe HIDA domain symptoms are more likely than those with other BPSD to be institutionalized and put in restraints (Matteson and Linton, 1996; Gilley et al., 2004). Further, a 2016 meta-analysis suggested these symptoms are common, revealing that individual symptoms within the HIDA domain occur at a prevalence rate of 17% to 40% in patients with AD (Fauth and Gibbons, 2014, Zhao et al., 2016). These symptoms are often more prevalent as patients can no longer be cared for at home, and rates of aggression and agitation may be as high as 60% in care facilities (Margallo‐Lana et al., 2001).

In this review, we focus on the HIDA domain from the likely pathophysiology to symptom management, including non-pharmacological and pharmacological interventions. Regarding treatment for the HIDA domain, most studies have focused on reducing aggression and agitation, with no or very few studies focusing on the treatment of aberrant motor activity, irritability, impulsivity, or disinhibition directly. Therefore, this review summarizes current trends in treating agitation and aggression specifically, noting the non-pharmacological and pharmacological trends in management as well as future directions that may yield newer strategies for improved patient care.

## Pathophysiology of the Hida Domain

Though the specific molecular mechanisms that lead to HIDA domain symptoms are generally unknown, pathology and human imaging studies have provided some insight into this domain’s pathophysiology. At first, the HIDA domain may seem like a disparate set of aberrant motor dysfunctions and behavioral states, but these symptoms all represent a common deficit in the appropriate inhibition of one’s actions. Mirroring this common deficit, very similar neurocircuitry is implicated for all of these symptoms, namely, loss of corticostriatal control and reduction in neurotransmission of far-reaching monoaminergic inputs that modulate this corticostriatal circuitry (Coccaro et al., 2011; Blair, 2016; Waltes et al., 2016; Dalley and Robbins, 2017). While specific investigation into the symptoms of the HIDA domain in AD has been limited, similar brain regions are usually implicated (Rosenberg et al., 2015).

More specifically, frontal cortical brain areas such as the orbitofrontal cortex, the ventromedial prefrontal cortex, and anterior cingulate cortex interact with the ventral and dorsal striatal nuclei, mediating inhibition of impulsive thoughts and motor responses, respectively (Coccaro et al., 2011; Whelan et al., 2012; Hoptman, 2015; Blair, 2016; Dalley and Robbins, 2017; Leclerc et al., 2018). In addition, areas like the amygdala, periaqueductal gray, anteroventral medial hypothalamus, lateral septum, ventral hippocampus, and medial preoptic nucleus promote impulses for certain behaviors (Coccaro et al., 2011; Hoptman, 2015; Blair, 2016; Dalley and Robbins, 2017; Leclerc et al., 2018). In terms of monoaminergic pathways, serotonin, norepinephrine, and dopamine are all implicated in these impulsive tendencies, and multiple genetic studies have implicated receptors and enzymes involved in these signaling pathways as influencing the presentation of these behaviors (Waltes et al., 2016). In AD, reductions in cholinergic and serotonergic markers have been reproducibly associated with agitation and aggression (Rosenberg et al., 2015). In terms of AD pathology, increased pS396 tau in Brodmann area 9 was associated with increased agitation and aggression (Guadagna et al., 2012), suggesting that increased phosphorylated tau in specific frontal cortical regions may lead to loss of inhibitory control over one’s actions.

Interestingly, the brainstem monoaminergic nuclei—especially the serotonergic dorsal raphe nucleus and noradrenergic locus coeruleus—are some of the first to degenerate in AD, suggesting that loss of normal function in these regions may underlie some symptoms within the HIDA domain in the early stages of the disease (Parvizi et al., 2001; Lyness, 2003; Šimić et al., 2017). Some degeneration in the mesolimbic dopamine circuitry has also been described and may contribute to these symptoms (Lyness, 2003; Nobili et al., 2017; D’Amelio et al., 2018). Comparatively, later spread of pathology to frontal cortical areas may lead to more severe symptoms within the HIDA domain at later stages of AD, as spread of AD pathology tends to progress from the ventral to dorsal cortical areas (Braak and Braak, 1991; Šimić et al., 2017). Though speculative, these represent two complementary pathways of neurodegeneration that may underlie HIDA domain symptoms.

## Non-Pharmacological Treatment Approaches

Dementia treatment guidelines assert that non-pharmacological approaches are first-line for mild or moderate HIDA domain symptoms that do not compromise the patient’s immediate safety (American Geriatrics Society and American Association for Geriatric Psychiatry, 2003). Unlike drug treatments for BPSD, which tend to carry a high risk of side effects for elderly patients, the side effects for psychosocial interventions are minimal (Sink et al., 2005; Konovalov et al., 2008; Coupland et al., 2011; Brodaty and Arasaratnam, 2012). There are an extensive number of non-pharmacological interventions available that vary greatly in their effectiveness in terms of target symptoms (Livingston et al., 2005; Azermai et al., 2012; Azermai, 2015; Tible et al., 2017; Legere et al., 2018). This section will describe how treatments rooted in psychological theory can be tailored towards patients with dementia to ameliorate HIDA domain symptoms. Moreover, it will outline a number of sensory stimulation and bodily activation techniques that are independent of theoretical orientation yet can be implemented to treat the HIDA domain as well. For a succinct overview of the non-pharmacological interventions discussed in this section, refer to **Table 1**.

**Table 1:** Non-pharmacological interventions for BPSD.

Person-centered and Behavioral Interventions			
Intervention	Definition	Studies	Efficacy
Person-Centered Care	Valuing patients with dementia, assessing and meeting their individual needs, viewing the world from their perspective, and promoting positive relationships and communications (Brooker, 2003)	Sloane et al., 2004 Fossey et al., 2006 Chenoweth et al., 2009	- Training programs are generally effective for reducing agitation, aggression, and antipsychotic use
Needs-Driven Approaches	Analyzing the context of patients’ BPSD to determine and address unmet needs (Cohen-Mansfield et al., 2007)	Kovach et al., 2006 Cohen-Mansfield et al., 2007 Husebo et al., 2011 Cohen-Mansfield et al., 2012	- Addressing patients’ overall unmet needs leads to significant reductions in agitation during the intervention period - Attending to pain needs specifically has mixed short-term efficacy on agitation with no evidence for long-lasting benefits
Validation Therapy	Encouraging patients with dementia to express their feelings and legitimatizing these communications regardless of quality or content (Toseland et al., 1997)	Toseland et al., 1997 Deponte and Missan, 2007	- Only one study on HIDA domain symptoms - No evidence for a significant benefit over other psychotherapies nor translation to clinical outcomes
Reminiscence Therapy	Using prompts to stimulate memories that patients have intact, allowing them to re-experience and share these memories (Tadaka and Kanagawa, 2007)	van Diepen et al., 2002 Tadaka and Kanagawa, 2007 Van Bogaert et al., 2016 O’Shea et al., 2014	- No evidence for that this therapy produces significant reductions in HIDA domain behaviors
Reality Orientation Therapy	Repeating orienting information (e.g., time, location, date, or weather) over a prolonged period of time each day to patients (Wallis et al., 1983)	Hanley et al., 1981 Wallis et al., 1983 Baldelli et al., 1993 Onder et al., 2005	- No evidence for efficacy in reducing overall BPSD - RCTs have not examined HIDA domain behaviors specifically
Simulated Presence Therapy (Family Presence Therapy)	Presenting patients with audiotapes or videotapes of loved ones recounting pleasant, autobiographical memories to increase the familiarity of the environment (Woods and Ashley, 1995)	Camberg et al., 1999 Garland et al., 2007	- Mixed efficacy findings for ameliorating HIDA domain - No evidence for lasting effects
Behavior Management	Identifying problematic behaviors and modifying the environment in ways that discourage them while promoting positive behaviors (Gormley et al., 2001)	Suhr, 1999 Teri et al., 2000 Gormley et al., 2001 Weiner et al., 2002 Burgio et al., 2002 Huang et al., 2003	- Therapist-conducted behavioral management significantly reduces overall BPSD - Informal, caregiver-conduced behavioral management generally does not produce significant, long-term reductions in HIDA domain symptoms
Cognitive Behavioral Therapy (CBT)	Addressing maladaptive interactions between thoughts, emotions, and behaviors *via* therapeutic experiences and coping skills tailored towards patients with dementia (Spector et al., 2015)	Marriott et al., 2000 Akkerman, 2004 Kurz et al., 2012 Kwok et al., 2014 Spector et al., 2015	- Generally effective in decreasing overall BPSD and symptoms within the affective domain, such as depression - Some evidence for long-lasting effects - No RCTs conducted on the HIDA domain
Staff and Informal Caregiver Psychoeducation	Educating caregivers or staff about dementia and BPSD, including how to cope with and manage stressful situations and problematic behaviors (Livingston et al., 2005)	McCallion et al., 1999 Hébert et al., 2003 Hepburn et al., 2007 Deudon et al., 2009	- Effective in reducing caregiver burden, overall BPSD, and agitation
Sensory stimulation interventions			
Intervention	Definition	Studies	Efficacy
Aromatherapy	Eliciting olfactory stimulation *via* essential oils with or without therapeutic touch (Yoshiyama et al., 2015)	Smallwood et al., 2001 Ballard et al., 2002 Holmes et al., 2002 Snow et al., 2004 Lin et al., 2007 Burns et al., 2011 Fu et al., 2013 O’Connor et al., 2013	- Inconsistent effect on agitation across studies with a trend towards insignificant or absent benefits
Massage or Therapeutic Touch	Applying pressure with the hands to certain parts of a patient’s body (e.g., hands or feet) using a slow, stroking motion (Suzuki et al., 2010)	Smallwood et al., 2001 Remington, 2002 Hicks-Moore and Robinson, 2008 Fu et al., 2013 Moyle et al., 2014	- Mixed findings regarding effect on agitation - No evidence for a significant benefit over other sensory stimulation approaches
Music Therapy	Listening or actively participating in music in a controlled therapeutic environment in order to accomplish treatment goals (Pedersen et al., 2017)	Remington, 2002 Garland et al., 2007 Raglio et al., 2008 Cooke et al., 2010 Sung et al., 2012 Sánchez et al., 2016b	- Most studies do not show a significant positive effect over other non-pharmacological approaches - Most studies do not provide evidence of long-term benefits
Light Therapy	Providing a source of artificial light for a period of time during the day or night	Ancoli-Israel et al., 2003 Dowling et al., 2007 Burns et al., 2009	- Ineffective in producing clinically significant reductions in agitation and hyperactivity in patients with dementia
Multisensory Stimulation Therapy/Snoezelen Therapy	Using various stimuli to activate multiple senses, facilitating patients’ interaction with their environment in a non-directive way that requires few intellectual and physically demands (Sánchez et al., 2016a)	Robichaud et al., 1994 Baker et al., 2001 van Diepen et al., 2002 Baker et al., 2003 Baillon et al., 2004 Sánchez et al., 2016a	- Mixed findings on ability to significantly reduce agitation and aggression - No evidence for a significant benefit over other sensory stimulation approaches - Most studies do not support long-term efficacy
Physical Activity	Usually walking and/or muscle training (Tible et al., 2017)	Alessi et al., 1999 Cott et al., 2002 Rolland et al., 2007 Eggermont et al., 2010 Lowery et al., 2014	- Most evidence shows that physical activity does not have a positive, significant impact on nighttime restlessness, daytime activity, irritability, or overall BPSD
Therapeutic Activities	Engaging patients in meaningful activities such as playing games, completing puzzles, or reading	Baker et al., 2001 Buettner and Fitzsimmons, 2002 Fitzsimmons and Buettner, 2002 Kolanowski et al., 2011	- Mixed findings regarding efficacy for reducing agitation - No evidence for lasting benefits

BPSD, behavioral and psychological symptoms of dementia; HIDA, hyperactivity–impulsivity–irritiability–disinhibition–aggression–agitation.

### Person-Centered Interventions

Person-centered care focuses on techniques that value dementia patients as individuals, assess and meet personal needs, view the world from patients’ perspectives, and facilitate positive relationships and communications (Brooker, 2003). Two randomized controlled trials (RCTs) exploring the effects of staff-training programs in person-centered care revealed that these programs resulted in significant reductions in patients’ agitation and aggression (Sloane et al., 2004; Chenoweth et al., 2009). Another RCT in which care staff received training in person-centered care demonstrated that this approach significantly reduced the extent to which patients were prescribed antipsychotics, a common agent used to treat HIDA domain symptoms (Fossey et al., 2006). However, there was no difference between groups in scores on the CMAI or in instances of aggression. This suggests that person-centered approaches may have modest benefits in reducing the severity of agitation and aggression, likely prolonging or negating the use of pharmacological alternatives.

Within the umbrella of person-centered care techniques, various approaches and therapies have been studied individually for reducing HIDA domain symptoms. One of these approaches is needs-driven care in which assessing and addressing patients’ needs is postulated to prevent agitation and aggression. In a needs-driven approach, care providers analyze the contexts in which disruptive behaviors occur to determine if these behaviors are an expression of patients’ unmet needs and seek to address them. Thus, a number of RCTs have examined the effects of needs-driven approaches in ameliorating BPSD (Kovach et al., 2006; Cohen-Mansfield et al., 2007; Husebo et al., 2011; Cohen-Mansfield et al., 2012). Some RCTs have found that broadly addressing patients’ unmet needs is associated with significant decreases in agitation during the intervention period (Cohen-Mansfield et al., 2007; Cohen-Mansfield et al., 2012). Studies examining the effects of addressing pain needs specifically have revealed mixed, short-term results in ameliorating general disruptive behaviors and symptoms of agitation (Kovach et al., 2006; Husebo et al., 2011). However, research has failed to demonstrate long-term, significant reductions in HIDA domain symptoms relative to usual care once needs-based interventions end (Husebo et al., 2011). Future studies should clarify which aspects and types of needs-driven care are most beneficial in ameliorating agitation and aggression in patients with dementia. Given that addressing unmet needs has generally delivered promising short-term results, future research should also focus on how to implement needs-driven approaches on a more continual basis to elicit long-term benefits (Cohen-Mansfield et al., 2007; Cohen-Mansfield et al., 2012; Husebo et al., 2011).

Promoting positive social interactions and meaningful relationships is also central to person-centered care. As dementia progressively compromises patients’ communication abilities, attempts to communicate may manifest as agitated and aggressive behaviors (Ragneskog et al., 1998). Validation therapy purports that BPSD have latent causes and that communications from patients with dementia are meaningful regardless of aberrant content or expression (Dietch et al., 1989). Patients can become withdrawn or agitated when they feel disregarded; thus, therapists performing validation therapy practice empathy towards patients with dementia, encourage them to express their feelings, and legitimatize these communications (Feil, 1982; Dietch et al., 1989; Toseland et al., 1997). There have not been many high-quality RCTs of validation therapy, and only one RCT has examined the impact of validation therapy on HIDA domain symptoms (Toseland et al., 1997). In comparison with social contact or with usual care, this intervention led to significant decreases in nurses’ ratings of patients’ physical and verbal aggression. However, these differences did not translate to other clinical outcomes such as frequency of physical restraint use, psychotropic delivery, or time spent intervening due to difficult behavior. Validation therapy, therefore, has the potential to reduce the perceived severity of HIDA domain symptoms, but there is no evidence yet that this intervention translates to other clinical outcomes nor superior to other non-pharmacological approaches. Thus, this approach may benefit from additional research aimed at identifying how and why benefits in clinical ratings fail to transfer into tangible outcomes.

Reminiscence therapy is a second approach that aims to promote positive social interactions and communications with patients who have dementia. It involves stimulating old memories with prompts such as photographs or songs, emphasizing intact cognitive abilities, and encouraging patients to share their memories with others (Tadaka and Kanagawa, 2007). One small RCT demonstrated that both validation therapy and reminiscence therapy significantly reduced overall BPSD scores as measured by the NPI in patients compared with patients receiving no treatment (Deponte and Missan, 2007). Moreover, reminiscence therapy led to significantly improved cognitive functioning and performance of activities of daily living in this study than did validation therapy. However, in RCTs specifically focusing on the HIDA domain, reminiscence therapy has not produced significant reductions in these behaviors nor significant increases in life quality (van Diepen et al., 2002; Tadaka and Kanagawa, 2007; O’Shea et al., 2014; Van Bogaert et al., 2016).

One of the first psychological therapies that drew on person-centered approaches for dementia patients was reality orientation therapy (Bowlby, 1991; Spector et al., 2000), which involves repeating orienting information, such as the date or the weather, over a prolonged period of time each day (Wallis et al., 1983). This approach aims to re-orient patients with dementia to their environments and to increase their engagement (Bowlby, 1991). The literature contends that reality orientation therapy should occur within a person-centered framework in which care providers facilitate positive, quality interactions with patients rather than simply providing information (Dietch et al., 1989). Likewise, case examples illustrate that reality orientation delivered mechanically may actually increase agitation and aggression in some patients with dementia (Dietch et al., 1989). RCTs utilizing reality orientation therapy have demonstrated mild, short-term cognitive improvements; however, they have not shown that it elicits a significant reduction in overall BPSD (Hanley et al., 1981; Wallis et al., 1983; Baldelli et al., 1993; Onder et al., 2005). Unfortunately, one frequent method of delivering reality orientation therapy utilizes a classroom approach (Bowlby, 1991), but patients who exhibit aggressive symptoms or wandering are often excluded due to concerns about managing disruptions in a classroom setting (O’Connell et al., 2007). Consequently, no studies have specifically examined the effects of reality orientation therapy on behaviors within the HIDA domain. In addition, despite the immediate popularity of reality orientation therapy following its creation in the mid-1960s, its use has decreased substantially in recent decades due to the formulation of other approaches (Spector et al., 2000).

Similar to reality orientation therapy, simulated presence therapy attempts to make the environment less foreign for patients with dementia. Simulated presence therapy—sometimes called family presence therapy—presents patients with audiotapes or videotapes of loved ones recounting pleasant, autobiographical memories (Woods and Ashley, 1995). As opposed to reminiscence therapy in which care providers use prompts to elicit memories and to facilitate communication, the goal of simulated presence therapy is to minimize distress by increasing the familiarity of the environment with the simulated presence of a family member. Some studies demonstrate significant yet short-lasting reductions in verbal and physical aggression during therapy sessions (Garland et al., 2007). However, other studies have failed to demonstrate similar reductions in patients’ agitation (Camberg et al., 1999). Thus, research findings regarding simulated presence therapy have been mixed, and there is no evidence that this intervention leads to long-term benefits.

### Behavioral Interventions

As described, person-centered care involves tailoring patients’ environments and treatments to align them better with individual values, needs, and perspectives. By increasing patients’ congruence with their surroundings, this approach aims to reduce BPSD by increasing patients’ quality of life, promoting positive feelings and experiences, and facilitating healthy social interactions and relationships. In contrast, the goal of behavioral interventions is to identify specific, problematic stimuli or situations that may be eliciting disruptive behaviors from a patient with dementia. Moreover, therapists teach caregivers and dementia patients specific strategies for lessening the frequency of these disruptive behaviors. Behavioral interventions for BPSD most prominently include behavior management and cognitive behavioral therapy (CBT).

Behavioral management strategies presume that disruptive behaviors in patients with dementia are due to maladaptive interactions between patients and their environment as a result of AD (Gormley et al., 2001). Thus, behavioral management techniques aim to identify the contexts in which problematic behaviors occur and to modify the environment to decrease the likelihood of these behaviors. One RCT focusing on therapist-conducted behavior management demonstrated significant reductions in overall BPSD as assessed with the NPI (Suhr, 1999). Behavioral management RCTs targeting the HIDA domain generally involve teaching caregivers to employ corresponding strategies in more informal settings (Teri et al., 2000; Gormley et al., 2001; Burgio et al., 2002; Weiner et al., 2002; Huang et al., 2003). Two of these studies showed some significant benefits of informal behavior management on agitation symptoms; however, there was no evidence that this effect persisted beyond intervention completion, and the majority of studies did not elicit similar positive results. Given the success of professionally administered behavior management on global BPSD, future studies examining the effects of formal behavior management on the HIDA domain may produce more positive results.

While behavioral management techniques focus solely on adjusting a patient’s environment to lessen the frequency of disruptive behaviors, CBT is more complex in that it teaches patients skills to address maladaptive interactions between their thoughts, emotions, and behaviors (Spector et al., 2015). Although CBT involves a learning component, there is evidence that some patients with dementia can acquire new skills with cognitive training despite impairment (Spector et al., 2003). Moreover, therapists can modify the content of the skills they teach and the methods they use to deliver them in ways that are compatible with the diminished cognitive function in dementia (Stanley et al., 2013). While several RCTs have shown that CBT can significantly decrease overall BPSD and lead to long-lasting reductions in affective domain symptoms, namely, depression (Marriott et al., 2000; Kwok et al., 2014; Spector et al., 2015), some research has not demonstrated that CBT has significant benefits on patients’ behavioral disturbances (Kurz et al., 2012). Additionally, there have been no studies that have directly implicated its use for aggression or agitation. Therefore, research into the efficacy of CBT in treating HIDA domain symptoms is needed.

Caregiver and staff psychoeducation targeted towards BPSD goes beyond education in person-centered care or behavioral interventions in that it provides information about dementia and effective coping, communication, and behavioral management strategies (Livingston et al., 2005). Although studies have examined the sole effects of psychoeducation programs on BPSD, elements of this approach relate to both person-centered and behaviorally oriented interventions and can, thus, be used in combination with the other non-pharmacological strategies described above. RCTs have consistently demonstrated that dementia-related psychoeducation reduces BPSD and perceived caregiver burden (McCallion et al., 1999; Hébert et al., 2003; Hepburn et al., 2007). Moreover, staff education programs can significantly reduce agitation among nursing home residents with dementia (Deudon et al., 2009). Thus, on an institutional level, psychoeducation of caregivers and staff is one of the most effective ways to reduce HIDA domain symptoms.

### Sensory Stimulation Interventions

Patients with dementia who are socially isolated, inactive, or bored demonstrate increased verbally disruptive behavior and excess motor activity (Cohen-Mansfield, 2000; Cohen-Mansfield et al., 2015). Thus, the goals of sensory stimulation techniques are to reduce these behaviors by increasing engagement and alertness (Strøm et al., 2016). These interventions can activate either a single sensory modality or multiple sensory modalities within a session. Approaches that use a sensory stimulation orientation include aromatherapy, massage, music therapy, light therapy, multisensory stimulation, physical activity, and therapeutic activities. Though head-to-head investigations of these techniques are sparse, and few if any of these techniques have been found to be beneficial in every study, certain approaches have more consistently demonstrated success in the literature than others.

For instance, studies of music therapy for agitation and aggression have shown mixed results. During music therapy, care providers utilize music in a controlled environment to facilitate the accomplishment of treatment goals (Pedersen et al., 2017). In the context of the HIDA domain, it is thought that music may reduce distress, promote quicker adaptation, and improve communication, thus reducing behaviors such as agitation and aggression that can arise when patients feel disoriented and frustrated in their attempts to communicate (Raglio et al., 2008). Active music therapy involves patients engaging in music activities such as singing or playing instruments, whereas passive music therapy involves patients listening to music. One RCT utilizing active music therapy showed that it significantly reduced dementia patients’ aberrant motor behaviors, irritability, and agitation, and these effects remained a month after intervention completion (Raglio et al., 2008). However, most other RCTs have not found that active nor passive music therapy reduces HIDA domain symptoms to a greater degree than other non-pharmacological interventions or control conditions. (Remington, 2002; Garland et al., 2007; Cooke et al., 2010; Sung et al., 2012; Sánchez et al., 2016b). Moreover, these RCTs do not provide evidence that music therapy can produce long-lasting effects. Still, music therapy may be helpful for particularly responsive patients, especially if other techniques have failed.

Studying the effects of aromatherapy on agitation has also produced mixed results, though findings trend towards insignificant benefits (Ballard et al., 2002; Holmes et al., 2002; Snow et al., 2004; Lin et al., 2007; Burns et al., 2011; O’Connor et al., 2013). To explain the heterogeneity in these results, some researchers hypothesize that essential oil odors alone may not be beneficial for patients with severe dementia, as their olfaction is likely to be greatly impaired (Snow et al., 2004). Rather, these researchers suggest that the non-specific elements of aromatherapy, such as touch and human interaction, may drive the positive effects seen in some research (Burns et al., 2011). Despite this notion, RCTs examining the effects of massage on agitation have also been mixed. While some have demonstrated that massage in combination with aromatherapy can produce mild benefits on agitation (Smallwood et al., 2001), other RCTs have not managed to corroborate this finding (Fu et al., 2013). Additionally, some RCTs have shown that massage alone can significantly reduce agitation than can no treatment (Remington, 2002; Hicks-Moore and Robinson, 2008), though others have actually demonstrated an increase in agitation following massage (Moyle et al., 2014). Similarly, there is no evidence that massage exerts greater benefit than other sensory stimulation interventions (Hicks-Moore and Robinson, 2008; Moyle et al., 2014).

Bright light therapy has become increasingly used to treat hyperactive delirium, as circadian dysfunction has been found to greatly impact delirium severity. In turn, bright light therapy was found to improve functional status and sleep in patients with hyperactive, perioperative delirium, which occurs more commonly in patients with dementia compared with otherwise healthy elderly patients (Chong et al., 2013). Despite its benefits for delirium, non-delirious patients with dementia do not show a reduction in agitation or aberrant motor symptoms with bright light therapy (Ancoli-Israel et al., 2003; Burns et al., 2009; Dowling et al., 2007). Even in studies in which symptom reductions were observed, light therapy rarely outperformed care as usual or placebo, and caregiver-perceived improvements were not noted (Ancoli-Israel et al., 2003; Burns et al., 2009). Moreover, one study in which light therapy did lead to a statistically significant improvement in agitation and aberrant motor behavior stressed that this improvement was not large enough to be clinically meaningful (Dowling et al., 2007).

In contrast to sensory stimulation-oriented approaches that focus on activating one sensory system at a time, multisensory stimulation therapy—also known as Snoezelen therapy—utilizes a variety of stimuli to activate multiple senses simultaneously (Sánchez et al., 2016a). As described previously, agitation and aggression may arise in patients with dementia who can no longer communicate effectively and who feel socially isolated. Thus, multisensory stimulation therapy is thought to reduce these symptoms by facilitating patients’ non-verbal interactions with their environment (Lykkeslet et al., 2014). Therapists conduct this intervention in a non-directive manner, promoting feelings of security in patients with dementia as they explore their environment in ways that require few intellectual and physical demands (Strøm et al., 2016; Sánchez et al., 2016a). Most RCTs comparing multisensory stimulation therapy with other approaches (e.g., individualized music sessions, reminiscence therapy, and structured activities) have demonstrated comparably significant reductions in agitation and aggression (Robichaud et al., 1994; Baker et al., 2001; Baker et al., 2003; Baillon et al., 2004; Sánchez et al., 2016a, Sánchez et al., 2016b), and similarly, these reductions rarely last beyond intervention completion (Baker et al., 2001; Baker et al., 2003; Sánchez et al., 2016a). Further, other RCTs have not demonstrated that multisensory stimulation therapy significantly reduces agitation or disruptive behavior (Robichaud et al., 1994; van Diepen et al., 2002). Thus, there is no evidence that multisensory stimulation therapy outperforms other sensory stimulation techniques that target one modality.

Although they do not specifically activate the senses, physical activity interventions targeting HIDA domain symptoms are very similar to sensory stimulation-oriented approaches in therapeutic philosophy. Physical activity programs for patients with dementia often involve light aerobic exercise and/or strength training to increase kinesthesia (Tible et al., 2017). While RCTs examining the effects of exercise on overall BPSD have not found significant, objective benefits (Rolland et al., 2007; Lowery et al., 2014), one study observed that perceived caregiver burden was significantly lower for patients who participated in a physical activity program (Lowery et al., 2014). Still, research regarding the impact of exercise training on HIDA domain symptoms in particular has not revealed significant, positive findings for patients (Alessi et al., 1999; Cott et al., 2002; Eggermont et al., 2010), and corresponding studies have not demonstrated that physical activity significantly ameliorates patients’ nighttime restlessness, daytime activity, or irritability.

Therapies that engage patients in meaningful activities such as playing games, completing puzzles, or reading aim to decrease agitation related to boredom and inactivity. One study in which dementia patients performed therapeutic activities revealed significant reductions in patients’ overall BPSD than in controls; however, these effects did not persist beyond intervention completion (Baker et al., 2001). Within the HIDA domain specifically, RTCs utilizing therapeutic activities have shown inconsistent benefits towards ameliorating agitation in patients with dementia (Kolanowski et al., 2011).

Overall, there is inconclusive evidence for the immediate benefits of sensory stimulation approaches during intervention sessions, and there is little to no evidence that these approaches can lead to long-lasting reductions in HIDA domain symptoms. Some have conceptualized sensory stimulation interventions as a way to alleviate agitation and aggression due to boredom or a lack of sensory stimulation (Cohen-Mansfield, 2013). Thus, it is logical that any benefits observed during sensory stimulation sessions will remit once those sessions end. Research regarding sensory stimulation approaches within the context of a broader needs-based or person-centered framework may elicit more consistent, lasting results.

## Pharmacological Treatment Approaches

As much of AD research has focused on mitigating cognitive decline, no drugs have been specifically designed to treat BPSD in AD. In fact, even among available medications, the FDA has yet to approve any of them for treatment of any BPSD in AD (Geda et al., 2013). Despite this lack of clear direction, clinicians routinely prescribe a number of common neuropsychiatric medications, especially for patients in long-term care facilities (Kirkham et al., 2017). Numerous professional societies—such as the American Psychiatric Association, the American Association for Geriatric Psychiatry, and the American Geriatrics Society—have suggested that non-pharmacological interventions are first-line therapy for BPSD, including HIDA domain symptoms (Kales et al., 2014; Reus et al., 2016; Lanctôt et al., 2017). Moreover, these organizations have recommended that pharmacological options should only be employed when a patient’s behaviors are severe or when non-pharmacological options have been tried and failed. The appropriate medications for HIDA domain symptoms specifically is still a topic of debate, and almost all meta-analyses and organization guidelines suggest that these pharmaceuticals are of modest benefit (Ballard and Corbett, 2010; Kales et al., 2014).

### Antipsychotics

The most commonly used class of drugs to treat HIDA domain symptoms are the atypical antipsychotics, with risperidone even being approved for this purpose in Europe, Canada, New Zealand, and Australia but not in the USA (Yunusa et al., 2019). Atypical antipsychotics share a common mechanism of action in reducing serotonin 2A receptor (5HT2A) activity, though they also provide some antagonism of other serotonergic receptors (5-HTRs) and the dopamine 2 receptor (D2R), similar to typical antipsychotics. Though risperidone is the best studied, other atypical antipsychotics may offer some benefits. For instance, a meta-analysis in 2011 concluded that there was high evidence to suggest that risperidone, aripiprazole, and olanzapine provide benefits for a total global outcome score of BPSD and HIDA domain symptoms specifically, including agitation and aggression (Maher et al., 2011). However, it was noted that the difference in total NPI scores was slightly below the threshold of minimum clinically significant change while the relative improvement was about 35%, just above the minimum clinically significant threshold (Maher et al., 2011). The extent to which changes in agitation and aggression specifically were clinically meaningful was not addressed. These results are largely in line with other meta-analyses of atypical antipsychotics for agitation and aggression in the context of dementia (Margallo‐Lana et al., 2001; Passmore et al., 2008; Ballard and Corbett, 2010; Kongpakwattana et al., 2018).

Even though the modest benefit of atypical antipsychotics has been demonstrated across multiple studies, the adverse effects of these medications represent serious risks that often outweigh the benefit of their implementation. This was most notably highlighted by the CATIE trial, a 42-outpatient-site and 421-AD-patient trial, which concluded that the risks posed by atypical antipsychotics outweigh the modest benefits in treating agitation, aggression, and psychosis in AD (Schneider et al., 2006). Since this time, other trials have echoed this narrow risk–benefit trade-off (Passmore et al., 2008; Maher et al., 2011). The most concerning adverse event to atypical antipsychotics is the increased risk of death in elderly populations, estimated to have an odds ratio of 1.7 and a number needed to harm (NNH) of 87 (Maher et al., 2011). In addition, the best-studied drug, risperidone, confers a three-fold higher risk of stroke with an NNH of 53, an increased risk of extra-pyramidal side effects at an NNH of 20, and an increased risk of urinary tract symptoms at an NNH of 16–36 (Maher et al., 2011). These concerns for heightened adverse effects—especially risk of death—resulted in a black box warning from the FDA for all atypical antipsychotics in elderly patients, and similar warnings have been issued in Europe and Canada (Koenig et al., 2016). In addition, many programs have aimed to discourage the use of antipsychotics in the elderly, including Beers criteria, Screening Tool of Older Persons’ Prescriptions (STOPP), Screening Tool to Alert Doctors to the Right Treatment (START), and Choosing Wisely (O’Mahony et al., 2015; Koenig et al., 2016; Kirkham et al., 2017; Yunusa et al., 2019). A recent Cochrane Systematic Review suggests that withdrawal from these medications has little effect or no effect on overall BPSD, mortality, and cognitive function (Leeuwen et al., 2018). Further, this review purports that discontinuing atypical antipsychotics after prolonged exposure may actually decrease agitation in patients displaying mild behavioral disturbances. Overall, the general recommendation from these studies is that if atypical antipsychotics are needed due to severe HIDA domain symptoms, there should be discussion of their risks and benefits and consideration of tapering after 4 months of use (Reus et al., 2016).

While these medications have their place in treating HIDA domain symptoms, determining the relative efficacy and safety of one atypical antipsychotic versus another has been challenging. In a recent network meta-analysis (Yunusa et al., 2019), aripiprazole was suggested to be the most effective compared with risperidone, olanzapine, quetiapine, and placebo, while risperidone and placebo had the lowest risk of death. In contrast, aripiprazole and quetiapine were safer in terms of cerebrovascular accidents (CVAs) compared with the higher risk of CVAs when taking risperidone or olanzapine. Overall, the authors concluded that no atypical antipsychotic could be singled out as being definitively more efficacious based on current evidence.

Regarding typical antipsychotics, most notably haloperidol, results of their efficacy for treating HIDA domain symptoms are mixed (Suh et al., 2006; Kongpakwattana et al., 2018; Jin and Liu, 2019). However, their higher risk for extrapyramidal side effects and mortality make them a poor choice for treating elderly patients, and they generally are considered to be less prudent in ameliorating HIDA domain symptoms than atypical antipsychotics (Ballard and Corbett, 2010; Reus et al., 2016).

The newest antipsychotic to receive Food and Drug Administration (FDA) approval, pimavanserin, works slightly differently than previous atypical antipsychotics through selective 5HT2A inverse agonism (Kitten et al., 2018). While approved to treat psychosis in Parkinson’s disease, the largest clinical trial of pimavanserin to treat AD-associated agitation and aggression showed no benefit of the drug at 6 to 12 weeks, though some secondary outcomes suggested modest benefit in treating irritability and emotional lability (Ballard et al., 2018).

### Antidepressants

Though this class of drugs encompasses numerous agents that alter reuptake of various monoamines, the selective serotonin reuptake inhibitors (SSRIs) are most studied for HIDA domain symptoms. Interestingly, these drugs are notoriously poor for treating affective domain symptoms in AD (Sepehry et al., 2012; Farina et al., 2017), suggesting that their manifestation is unlikely to be similar to that in otherwise healthy, young patients. However, these drugs may be prescribed at rates of 25–42% in patients with dementia (Farina et al., 2017), either owing to their effectiveness in treating agitation and other HIDA domain symptoms or reflecting the limited toolbox that physicians have in treating affective domain symptoms.

Similar to atypical antipsychotics, antidepressants likely provide only a minimal benefit for patients with HIDA domain symptoms (Seitz et al., 2011; Wilkins and Forester, 2016; Farina et al., 2017). Arguably, the best evidence for the roles of SSRIs in the treatment of agitation comes from the CIT-AD trial (Porsteinsson et al., 2014) in which citalopram was shown to reduce agitation symptoms on the Neurobehavioral Rating Scale (NRBS-A), Clinical Global Impression of Change (CGIC), and CMAI over 9 weeks. However, enthusiasm for the use of citalopram to treat HIDA domain symptoms was tempered by the relatively high dose of this medication used in the study (30 mg). Recent FDA guidelines suggest that doses above 20 mg may be dangerous for patients over 60 years of age due to significant QT prolongation (Farina et al., 2017). In addition, the study suggested increased rates of anorexia, diarrhea, fever, and worsening cognition in patients taking citalopram (Porsteinsson et al., 2014), which need to be considered when determining the risk–benefit trade-off for this medication.

The effects of citalopram on reducing agitation in AD likely extend to other SSRIs, supported by a 2011 Cochrane Review (Seitz et al., 2011), suggesting that antidepressants were superior to placebo in treating agitation in dementia. However, while the review implied that citalopram and sertraline had the best evidence, it was unable to comment on the differences in efficacy between them or other antidepressants, and current evidence suggests that antidepressants are equivocal to atypical antipsychotics in terms of efficacy in reducing agitation (Seitz et al., 2011; Wilkins and Forester, 2016). Overall, the use of SSRIs to treat HIDA domain symptoms as an alternative to atypical antipsychotics is promising and may confer lower risks of mortality, stroke, and motor side effects.

There is little evidence for newer antidepressants that target individual 5-HTRs in treating HIDA domain symptoms. For instance, mirtazapine was associated with some benefits for reducing agitation in a 12-week prospective cohort study, but RCTs have yet to be completed (Cakir and Kulaksizoglu, 2008). Similarly, trazodone has shown modest benefits in some cohort studies, but RCTs have not reproduced these findings (Seitz et al., 2011; Farina et al., 2017).

### Antiepileptic Drugs

The evidence for the use of antiepileptic drugs, compared with atypical antipsychotics and antidepressants, to treat HIDA domain symptoms remains scarce. The best evidence for antiepileptic drugs exists for valproic acid (VPA; also known as divalproex), for which a recent Cochrane Review of five RCTs suggested that the drug was probably ineffective for treating agitation in dementia (Baillon et al., 2018). Comparatively, a few very small RCTs (combined *n* < 100) implied a modest benefit of carbamazepine, though some trials showed no benefits (Gallagher and Herrmann, 2014). In addition, a meta-analysis supported the efficacy of carbamazepine for treating agitation in AD, but a 103-person RCT did not find a statistically significant benefit (Ballard et al., 2009)

Other antiepileptic drugs are much less well studied, usually having only case reports or small RCTs to support or discourage their use. For instance, gabapentin has a handful of case reports suggesting that it may be effective in treating agitation and aggression in dementia but has no prospective cohorts or RCTs (Supasitthumrong et al., 2019). Lamotrigine similarly has low-quality evidence from a retrospective chart review and open-label clinical trial showing that it may reduce agitation in dementia (Ng et al., 2009; Suzuki and Gen, 2015). Results for levetiracetam are mixed across two open-label studies of its effect on agitation and manic-like symptoms in the context of BPSD (Weiner et al., 2005; Kyomen et al., 2007). A retrospective chart review of 15 patients suggested some benefits of topiramate for aggression in dementia (Fhager et al., 2003), and a small RCT of 48 patients suggested a similar benefit of topiramate to risperidone in reducing agitation (Mowla and Pani, 2010). Finally, a single RCT of oxcarbazepine, compared with placebo, did not find differences in agitation or aggression (Sommer et al., 2009). Overall, increased investigation of antiepileptic drugs is warranted, and use of them in patients with severe HIDA domain symptoms and resistance to atypical antipsychotics or antidepressants may be warranted in select clinical situations.

### Cognitive Enhancers

Though cognitive decline in AD cannot currently be slowed or reversed, select drugs targeting cholinergic and glutamatergic pathways have been prescribed to ameliorate cognitive symptoms in this disease. The best-known drugs in this class are the acetylcholinesterase inhibitors and memantine, an NMDA antagonist. In general, acetylcholinesterase inhibitors are often used during the milder stages of AD, while memantine is often given as AD progresses to more moderate or severe stages. Because these drugs are often prescribed to patients with AD and other dementias, they are also well studied in terms of their effects on the HIDA domain, though often as secondary or exploratory analyses of larger trials.

Regarding acetylcholinesterase inhibitors, evidence for their efficacy in treating HIDA domain symptoms remains inconsistent. For instance, two meta-analyses have suggested that acetylcholinesterase inhibitors like donepezil may provide moderate benefits in reducing BPSD (Birks, 2006; Lockhart et al., 2011). However, an RCT of 272 patients did not find that donepezil reduced agitation or total NPI score (Howard et al., 2007). Finally, a systematic review of three acetylcholinesterase inhibitors suggested that only 3 out of the 14 included RCTs demonstrated a benefit of any acetylcholinesterase inhibitors on total NPI scores, agitation, or aggression, and the effect sizes were just at or below the minimum threshold for clinical significance (Rodda et al., 2009).

Results for memantine are similar. A recent Cochrane Review (McShane et al., 2019) suggested high-certainty evidence from 14 trials including 3,700 patients that memantine improves performance on the Clinical Global Ratings Scale (CGR), cognitive function, performance on the Severe Impairment Battery (SIB), and total NPI scores. However, the review concluded that while agitation occurred at a lower rate for patients treated with memantine, the drug provided no benefits when used to treat agitation specifically. Overall, the study suggested that although memantine was moderately effective in treating cognitive symptoms, it was unlikely to be an appropriate monotherapy for agitation. In conclusion, while acetylcholinesterase inhibitors and memantine may have small benefits on HIDA domain symptoms when used for their initial indications, it is unlikely they will provide much added benefits as monotherapy for HIDA domain symptoms.

### Other Pharmacological Agents

Most other studied drugs to treat the HIDA domain symptoms have scant evidence to support their use, and even fewer have undergone testing in RCTs. However, there are a few promising candidates that deserve to be investigated in more detail.

One of these is dextromethorphan–quinidine (AVP-923), a combination drug acting on multiple receptors, including NMDA antagonism, σ1 receptor agonism, serotonin and norepinephrine reuptake inhibition, and nicotinic α3β4 receptor antagonism. In the USA and Europe, this drug has already been approved to treat pseudobulbar affect in amyotrophic lateral sclerosis (ALS). Excitingly, a recent RCT of 194 AD patients over 10 weeks (Cummings et al., 2015) demonstrated that AVP-923 improved aggression and agitation scores as measured by the NPI and was similarly effective at different stages during the disease. In addition, secondary analyses suggested that the drug effectively lowered irritability and emotional lability, aberrant motor behavior, and caregiver strain as measured by the NPI. In conjunction, a recent network meta-analysis suggested that risperidone and AVP-923 were the only two drugs to reach significance for treating dementia-related agitation (Kongpakwattana et al., 2018). Though longer-term follow-up is needed to evaluate the full potential of AVP-923, this drug may represent another tool for physicians to use in treating HIDA domain symptoms.

Drugs that target noradrenergic receptors are another set of medications that have been suggested for treating HIDA domain symptoms. For instance, prazosin, an α1 receptor blocker, successfully decreased agitation and aggression symptoms in 22 nursing home or community-dwelling adults with AD (Wang et al., 2009). In addition, there are a few scarce reports and one small trial of the beta-blockers propranolol and pindolol demonstrating that these drugs can significantly reduce dementia-related agitation (Peskind et al., 2005; Passmore et al., 2008). Further investigation of these drugs in larger RCTs would be helpful.

Cannabinoids have also been tried in the treatment of HIDA domain symptoms. Specifically, while two RCTs studying THC found no benefits of the compound in treating agitation or other BPSD symptoms as measured by the NPI, five trials of dronabinol demonstrated a reduction in agitation, motor activity, and total NPI score (Sherman et al., 2018). As cannabinoid research continues to progress, it will be interesting to see if these medications truly benefit patients with HIDA domain symptoms.

Lastly, the atypical anxiolytics tandospirone and buspirone, which block 5HT1A, have been suggested as potential treatments for the HIDA domain. Specifically, there have been a few case reports (Passmore et al., 2008) and one retrospective study (Santa Cruz et al., 2017) of buspirone that have implied its efficacy in reducing agitation and aggression in dementia. Similarly, an open-label study of 13 dementia patients treated with tandospirone suggested reduction in NPI scores corresponding to delusions, agitation, depression, anxiety, and irritability at 2 and 4 weeks after administration (Sato et al., 2007). As the side-effect profiles of these drugs are particularly mild, RCTs with these drugs in treating HIDA domain symptoms would be beneficial.

### Antipsychotics and Histone Deacetylase (HDAC) Inhibitors: a Novel Approach With Epigenetics

Though other approaches to reduce HIDA domain symptoms are being investigated, first-line pharmacotherapy for these symptoms is still likely to be atypical antipsychotics. However, the severity of adverse effects in elderly patients appropriately gives many physicians pause in prolonged prescribing of these medications. Biologically, both aging and AD are known to result in a number of epigenetic alterations, and within the central nervous system (CNS), many of these alterations lead to a more repressive transcriptional environment, reducing expression of key receptors that atypical antipsychotics target (Mastroeni et al., 2010; Zhang et al., 2012; Akbarian et al., 2013; Cacabelos and Torrellas, 2015; McClarty et al., 2018). As patients age, the efficacy of atypical antipsychotics decreases while the frequency and severity of adverse effects increase, effectively leading to lower doses of medications prescribed but also less benefit at those doses, thus narrowing the therapeutic window (Schneider et al., 2006; Passmore et al., 2008; Maher et al., 2011). We hypothesized that aging-related histone deacetylation at certain gene promoter regions decreases the expression and functioning of drug-targeted receptors, therefore limiting this window in elderly patients. This was corroborated by studies showing that elderly patients had reduced expression and occupancy of D2R and 5-HT2AR *via* positron emission tomography (PET) and single-photon emission computed tomography (SPECT) imaging (Antonini et al., 1993; Versijpt et al., 2003).

Our own preclinical studies in aged and young mice support our hypothesis. Specifically, we showed that during aging, certain lysine residues on histones 3 and 4 become hypoacetylated at the *Drd2* promoter, leading to reduced D2R expression and greater sensitivity to extrapyramidal side effects of haloperidol (Montalvo-Ortiz et al., 2017). Importantly, we also showed that HDAC inhibition *via* VPA or entinostat (MS-275) can reverse the repressive histone marks, increase D2R expression, and reverse the aging-related sensitivity to haloperidol (Montalvo-Ortiz et al., 2017). Additionally, we demonstrated that c-Fos expression in response to antipsychotics in the frontal cortex could be modified by these HDAC inhibitors, thus suggesting that the efficacy of antipsychotics could be impacted by histone modifications with aging (Montalvo-Ortiz et al., 2014). In non-aged mice, there is evidence that chronic administration of atypical antipsychotics leads to 5-HT2AR-mediated repression of histone modifications at the mGlu2 promoter (Kurita et al., 2012). Kurita and colleagues demonstrated that combined administration of the HDAC inhibitor vorinostat (suberoylanilide hydroxamic acid (SAHA)) with clozapine or risperidone rescued 5-HT2AR-mediated repression of histone modification at the mGlu2 promoter and attenuated schizophrenia-like behavior. Whether repressed histone modification at the mGlu2 promoter primarily affects the efficacy of atypical antipsychotics in aged mice has yet to be shown. However, this study provides evidence that general alterations in histone modification are likely at play.

Similar to healthy aging, AD is also associated with various epigenetic changes that are thought to exacerbate pathological processes while simultaneously repressing nonpathological processes, thus contributing to disease progression (Mastroeni et al., 2010; Zhang et al., 2012). Concordantly, imaging studies have demonstrated significant reductions in 5-HT2AR expression and binding in patients with various stages of AD than in age-matched controls (Versijpt et al., 2003; Hasselbalch et al., 2008; Marner et al., 2012). It is, therefore, likely that in patients with AD, the effects of epigenetic changes associated with aging are compounded by the disease process itself, significantly reducing the availability of receptors on which atypical antipsychotics act. As a result, HDAC inhibition may have a dual effect in reducing the adverse side effects of long-term atypical antipsychotics and improving other symptoms of AD pathogenesis.

A number of studies have already examined monotherapy with HDAC inhibition in various preclinical mouse models of AD, revealing that HDAC inhibitors can improve memory performance in these mice (Francis et al., 2009; Corbett et al., 2017; Cuadrado-Tejedor et al., 2017; Cao et al., 2018). Interestingly, HDAC inhibitors are also being explored in clinical trials as monotherapy for cognition in dementia (Teijido and Cacabelos, 2018). Studies regarding the efficacy of HDAC inhibition in the context of BPSD specifically are limited, however, and findings are, therefore, inconclusive. Some research indicates that HDAC inhibitors do not ameliorate anxiety-like behavior and hyperactivity in mouse models of AD (Cao et al., 2018), whereas other studies demonstrate reduced hyperactivity and apathy-like behavior (Zhang and Schluesener, 2013; Selenica et al., 2014; Cathomas et al., 2015). It is worth noting that these studies have only investigated the effects of HDAC inhibition alone. There have yet to be any studies in which HDAC inhibitors are used in combination with other treatments such as antipsychotics in the context of BPSD, which warrants future research. Though it is unclear if HDAC inhibitors may directly be prescribed to treat the HIDA domain or other BPSD, it is an intriguing hypothesis that the addition of these drugs to atypical antipsychotics may result in increased efficacy and reduced side effects. As reversal of the extrapyramidal side effects has been the focus of current studies, investigation into the effects of these compounds on antipsychotic-induced cerebrovascular dysfunction in aged mice or patients may be especially promising.

## Beyond Agitation and Aggression

As evidenced in this review, most studies targeting the HIDA domain have mainly focused on agitation and aggression. Only a handful of quality trials have examined effects on patients’ aberrant motor activity or irritability (Cott et al., 2002; Dowling et al., 2007; Raglio et al., 2008; Cummings et al., 2015; Ballard et al., 2018; Sherman et al., 2018), and additional studies are needed in order to determine which approaches are most efficacious in treating these symptoms. Within the HIDA domain, RCTs of interventions for impulsivity and disinhibition are severely lacking. Similarly, there have been no RCTs assessing the efficacy of specific pharmacological or non-pharmacological approaches in treating these symptoms in AD (Tucker, 2010; Cipriani et al., 2016).

The discussion of treatment strategies to mitigate general disinhibition or impulsive behavior in AD patients within the literature is essentially nonexistent. Thus, current suggestions for treatment of these symptoms within this population are based on studies of these symptoms in other forms of dementia, such as frontotemporal dementia (FTD). One small, unblinded trial demonstrated that 67% of FTD patients with disinhibition who received SSRIs—namely, paroxetine, sertraline, and fluoxetine—experienced reductions in this symptom (Swartz et al., 1997). However, the authors did not report on the statistical significance of these reductions. Other small, open-label drug trials have demonstrated that treatment with SSRIs, such as citalopram and trazodone, can lead to significant reductions in disinhibition in patients with FTD (Lebert and Pasquier, 1999; Herrmann et al., 2012). Regarding potentially inefficacious pharmacotherapy for treating disinhibition, one study found that FTD patients treated with donepezil tended to demonstrate increased socially disinhibited behavior, such as inappropriate remarks or unusual interactions with strangers (Mendez et al., 2007). In the case of non-pharmacological interventions, the literature examining treatments for disinhibition and impulsivity in the context of dementia is even more scarce. However, case examples demonstrate that having patients with FTD engage in old hobbies or games may attenuate socially inappropriate, disinhibited behavior, potentially utilizing a similar mechanism as therapeutic activities that target agitation and aggression by reducing boredom and inactivity (Ikeda et al., 1995).

One common form of disinhibited behavior in AD is sexual disinhibition, defined as sexually oriented, verbal or physical acts that are inappropriate within the contexts that they are performed (Johnson et al., 2006). Regarding non-pharmacological methods to mitigate sexual disinhibition, the literature suggests redirecting behavior, expressing its inappropriateness, substituting staff who are less likely to trigger it, ignoring inappropriate and reinforcing appropriate behaviors, and providing patients with certain clothing that limits the likelihood of these behaviors (e.g., clothing that opens from the back; Kamel and Hajjar, 2003). In terms of pharmacotherapy, case studies and small, unblinded trials suggest that SSRIs—namely, citalopram—may have the potential to reduce sexual disinhibition in patients with dementia (Tosto et al., 2008). Case studies also suggest that the anticholinesterase inhibitor rivastigmine might be helpful for this symptom, whereas donepezil might increase sexual disinhibition (Alagiakrishnan et al., 2003; Lo Coco and Cannizzaro, 2010). Antiepileptics such as gabapentin and carbamazepine may ameliorate sexually disinhibited behavior in some patients with dementia (Miller, 2001; Alkhalil et al., 2004; Freymann et al., 2005). Regardless of these interventions’ potential, substantial additional research is needed before reliable conclusions about efficacy and recommendations for the treatment of sexual disinhibition in dementia can be made.

## Conclusions and Future Directions

### Summary of Effective and Promising Interventions for HIDA Domain Symptoms

Research findings suggest that the best non-pharmacological treatments for HIDA domain symptoms include training programs in person-centered care for staff and psychoeducation for staff and caregivers (McCallion et al., 1999; Hébert et al., 2003; Sloane et al., 2004; Fossey et al., 2006; Hepburn et al., 2007; Chenoweth et al., 2009; Deudon et al., 2009). Approaches that holistically assess and address patients’ unmet needs have shown to have significant benefits during periods of intervention, warranting future studies of how to implement these approaches long-term on a continuous basis (Cohen-Mansfield et al., 2007; Cohen-Mansfield et al., 2012). Although studies have not specifically examined the benefits of CBT on HIDA domain symptoms, evidence for lasting reductions in overall BPSD and affective domain symptoms calls for additional research into the efficacy of CBT for agitation an aggression (Marriott et al., 2000; Akkerman, 2004; Kurz et al., 2012; Kwok et al., 2014; Spector et al., 2015). Unlike certain person-centered and behavioral interventions, sensory stimulation interventions either have mixed evidence or have been shown to be generally ineffective in ameliorating HIDA domain symptoms (Robichaud et al., 1994; Alessi et al., 1999; Baker et al., 2001; Smallwood et al., 2001; Buettner and Fitzsimmons, 2002; Fitzsimmons and Buettner, 2002; Ballard et al., 2002; Holmes et al., 2002; Cott et al., 2002; Remington, 2002; van Diepen et al., 2002; Ancoli-Israel et al., 2003; Baker et al., 2003; Baillon et al., 2004; Snow et al., 2004; Dowling et al., 2007; Garland et al., 2007; Lin et al., 2007; Rolland et al., 2007; Hicks-Moore and Robinson, 2008; Raglio et al., 2008; Burns et al., 2009; Cooke et al., 2010; Eggermont et al., 2010; Burns et al., 2011; Kolanowski et al., 2011; Sung et al., 2012; Fu et al., 2013; O’Connor et al., 2013; Lowery et al., 2014; Moyle et al., 2014; Sánchez et al., 2016a; Sánchez et al., 2016b). However, studying sensory stimulation in the context of person centered or needs driven may produce more beneficial results.

Regarding pharmacotherapy, atypical antipsychotics are most commonly prescribed to treat HIDA domain symptoms, but their long-term use in elderly patients is severely limited due to an increased risk of extrapyramidal side effects, stroke, and death (Margallo‐Lana et al., 2001; Schneider et al., 2006; Passmore et al., 2008; Ballard and Corbett, 2010; Maher et al., 2011; Koenig et al., 2016; Kongpakwattana et al., 2018). Moreover, there is poor consensus as to which atypical antipsychotic is most efficacious (Yunusa et al., 2019). For antidepressants, SSRIs are the most common type prescribed for agitation and aggression in patients with dementia, but like atypical antipsychotics, they are only modestly effective in treating HIDA domain symptoms (Seitz et al., 2011; Porsteinsson et al., 2014; Wilkins and Forester, 2016; Farina et al., 2017). Still, it may be more beneficial to prescribe SSRIs than atypical antipsychotics in elderly patients because the risks of mortality, stroke, and extrapyramidal side effects are lower. However, whether the effects of these drugs are helpful for long-term control of HIDA domain symptoms is unclear. The evidence for other pharmacological approaches either does not support their use, as is the case for VPA, or is in its infancy, as is the case for AVP-923 (Fhager et al., 2003; Weiner et al., 2005; Birks, 2006; Howard et al., 2007; Kyomen et al., 2007; Ballard et al., 2009; Ng et al., 2009; Rodda et al., 2009; Sommer et al., 2009; Mowla and Pani, 2010; Lockhart et al., 2011; Gallagher and Herrmann, 2014; Cummings et al., 2015; Suzuki and Gen, 2015; Baillon et al., 2018; Kongpakwattana et al., 2018; Sherman et al., 2018; McShane et al., 2019; Supasitthumrong et al., 2019). Overall, substantial improvement is needed to increase the efficacy and reduce the side effects of pharmacotherapies for HIDA domain symptoms.

### Improving Treatment Implementation

Despite the fact that most professional geriatric medicine organizations recommend implementation of non-pharmacological therapies for BPSD first, medications are disproportionately favored to manage BPSD in clinical settings. One study revealed that in comparison with 71% of elderly nursing home residents who received pharmacotherapy, only 12% received non-pharmacological treatment (Molinari et al., 2010). Moreover, another study found that less than half of nursing home patients receiving antipsychotics had been prescribed in compliance with nursing home standards, and many were taking more than the maximum recommended dose or did not meet adequate symptom criteria for the prescription (Briesacher et al., 2005).

One reason for the disproportionately low use of non-pharmacological interventions for BPSD in real-world clinical settings is that properly assessing symptoms and implementing these treatments are time-consuming, and facilities often do not have adequate personnel (Kales et al., 2014). Similarly, research findings suggest that about half of nursing homes do not feel that they receive adequate psychiatric consultation, specifically in regard to non-pharmacological approaches (Reichman et al., 1998). It is also more difficult to get non-pharmacological interventions, compared with pharmacotherapy, reimbursed by insurance (Kales et al., 2014).

One of the most prominent reasons that non-pharmacological approaches are not implemented more frequently in clinical settings is the ambiguity that exists about which treatments to use and how to implement them. Methods are not standardized among non-pharmacological interventions of the same type, causing difficulty in interpreting results and in applying treatments in clinical practice (Leone et al., 2009). As a result, guidelines do not agree on their recommendations for specific psychosocial therapies and differ in respect to the quality of empirical support behind their guidance (Azermai et al., 2012). This leaves caregivers and clinicians with the burden of deciding which interventions are best. As there is no clear consensus on this, many physicians lack proper training in non-pharmacological approaches, BPSD assessment, and ways of choosing and communicating these interventions to caregivers (Kales et al., 2014).

To strengthen the consensus regarding non-pharmacological interventions, some researchers assert that evidence-based protocols and additional studies with greater methodological quality, particularly large RCTs, are necessary (Livingston et al., 2005; Kong et al., 2009; Vernooij-Dassen et al., 2010; Azermai, 2015; Scales et al., 2018). However, given that person-centered approaches tailored to individual patients appear to be the most efficacious, others have questioned the extent to which non-pharmacological approaches can be standardized and studied in large RCTs (Cohen-Mansfield, 2013).

To reconcile both of these perspectives and to integrate the implementation of both pharmacological and non-pharmacological interventions in practice, Kales and colleagues developed the describe, investigate, create, evaluate (DICE) approach (Kales et al., 2014). It involves detailed characterization of a patients’ BPSD and the context in which they occur, exploration and identification of potential underlying causes for disruptive behaviors, collaboration with both caregivers and patients to develop and implement a treatment plan, and evaluation of the extent to which a treatment plan was carried out and was effective. Future research may utilize the DICE approach to conduct controlled trials that differentiate non-pharmacological strategies on their most efficacious attributes without sacrificing the individualized component of treatment. Additionally, the DICE approach’s detailed symptom characterization and treatment evaluation may help to elucidate which patients will benefit most from certain pharmacotherapies.

To address concerns regarding inadequate resources in clinical settings for characterizing symptoms, implementing non-pharmacological interventions, and evaluating treatment responses, the literature suggests the use of environmental and wearable sensors (Bharucha et al., 2009; David et al., 2010; Kikhia et al., 2015). By continuously measuring patients’ behavior, these sensors are capable of detecting changes from routine and of collecting much more data than is possible with a limited nursing home staff or a single caregiver (Kikhia et al., 2015). Regarding side effects to pharmacotherapies, wearable sensors that measure patients’ vital signs and metabolic parameters could aid in early detection and intervention before the impact of these side effects becomes catastrophic (Bharucha et al., 2009). Although these methods are still being developed and are not yet widely used in clinical settings, research suggests that implementing them is feasible and well tolerated by patients with dementia. For instance, one study demonstrated that a nighttime monitoring system resulted in an 85% reduction in patients’ likelihood to have a dangerous event (e.g., injury due to wandering) due to aberrant nighttime behavior (Rowe et al., 2009). Moreover, this intervention was so well tolerated by patients and their caregivers that all who received it opted to continue using it following the completion of the study. Other research has demonstrated that actigraphy—the use of a small, unobtrusive accelerometer to measure motor activity—is a feasible and inconspicuous method that can indirectly and objectively measure the timing and frequency of various BPSD, including agitation and aberrant motor activity (Volkers et al., 2003; Mahlberg et al., 2007; David et al., 2010; Mulin et al., 2011). Overall, ambient and wearable sensors represent a promising way to improve the implementation of both pharmacological and non-pharmacological interventions for BPSD.

### Conclusion

Among various BPSD experienced by the majority of AD patients, HIDA domain symptoms are particularly tough to manage, causing great burden on caregivers and hospital staff and demonstrating an area of special concern regarding safety (Fuh et al., 2001; Rymer et al., 2002; Nguyen et al., 2008; Fauth and Gibbons, 2014). Difficulty controlling HIDA domain symptoms is compounded by the fact that currently available non-pharmacological and pharmacological interventions are only moderately efficacious. Despite the extensive toolbox of non-pharmacological treatments available, limited resources and guidance have impeded the extent to which they are used in real-world clinical settings (Kales et al., 2014).

Regarding pharmacotherapy, agents that are currently available are either inefficacious or carry the risk for dangerous side effects that outweigh their benefits (Margallo‐Lana et al., 2001; Schneider et al., 2006; Passmore et al., 2008; Ballard and Corbett, 2010; Maher et al., 2011; Koenig et al., 2016; Kongpakwattana et al., 2018). However, short-term use of these agents during periods of severe symptoms or psychosis may be necessary, and atypical antipsychotics and SSRIs currently have the best evidence. Newer approaches, including dextromethorphan–quinidine, cannabinoids, and HDAC inhibitors, are promising but will need more rigorous testing in RCTs before determining their use in these patients.

Future research utilizing the DICE approach to perform controlled trials on non-pharmacological interventions may be able to better differentiate non-pharmacological strategies on their most efficacious attributes while also providing individualized treatment (Kales et al., 2014). Moreover, future pharmacological studies that perform detailed symptom characterization and treatment evaluation *via* the DICE approach are more likely to reveal which patients will benefit most from certain pharmacotherapies. To further improve symptom characterization and treatment implementation in the real world, studies should also focus on bettering environmental and wearable sensors to make them more accessible to patients in clinical settings (Bharucha et al., 2009; David et al., 2010).

## Author Contributions

RK, DF, and HD decided upon the outline for this manuscript. RK and DF reviewed the literature extensively. All authors contributed to the writing and approval of this manuscript.

## Funding

This work was supported by the National Institute of Mental Health (1R01AG062249 and R01MH109466 to HD and 5F30MH109249-02 to DF).

## Conflict of Interest

The authors declare that the research was conducted in the absence of any commercial or financial relationships that could be construed as a potential conflict of interest.
